# Mental health inequalities in Slovenian 15-year-old adolescents explained by personal social position and family socioeconomic status

**DOI:** 10.1186/1475-9276-13-26

**Published:** 2014-03-28

**Authors:** Helena Jeriček Klanšček, Janina Žiberna, Aleš Korošec, Joca Zurc, Tit Albreht

**Affiliations:** 1 National Institute of Public Health, Trubarjeva 2, 1000 Ljubljana, Slovenia; 2 College of Nursing Jesenice, Sp. Plavž 3, 4270 Jesenice, Slovenia

**Keywords:** Slovenia, Socioeconomic position, Adolescents, Mental health, Inequalities

## Abstract

**Introduction:**

Mental health inequalities are an increasingly important global problem. This study examined the association between mental health status and certain socioeconomic indicators (personal social position and the socioeconomic status of the family) in Slovenian 15-year-old adolescents.

**Methods:**

Data originate from the WHO-Collaborative cross-national ‘Health Behavior in School-aged Children’ study conducted in Slovenia in 2010 (1,815 secondary school pupils, aged 15). Mental health status was measured by: KIDSCREEN-10, the Strength and Difficulties questionnaire (SDQ), a life satisfaction scale, and one question about feelings of depression. Socioeconomic position was measured by the socioeconomic status of the family (Family Affluence Scale, perceived material welfare, family type, occupational status of parents) and personal social position (number of friends and the type of school). Logistic regression and a multivariate analysis of variance (MANOVA) were performed.

**Results:**

Girls had 2.5-times higher odds of suffering feelings of depression (p < 0.001), 1.5-times higher odds of low life satisfaction (p = 0.008), and a greater chance of a lower quality of life and a higher SDQ score than boys (p = 0.001). The adolescents who perceived their family’s material welfare as worse had 4-times higher odds (p < 0.001) of a low life satisfaction, a greater chance of a low quality of life, and a higher SDQ score than those who perceived it as better (p < 0.001). Adolescents with no friends had lower KIDSCREEN-10 and higher SDQ scores than those who had more than three friends.

**Conclusions:**

Despite the fact that Slovenia is among the EU members with the lowest rates of social inequalities, it was found that adolescents with a lower socioeconomic position have poorer mental health than those with a higher socioeconomic position. Because of the financial crisis, we can expect an increase in social inequalities and a greater impact on adolescents’ mental health status in Slovenia in the future.

## Introduction

Mental health determines and is determined by a wide and diverse network of personal (e.g. genetic, lifestyle, psychological), social and community-related (e.g. family structure, friends, isolation), larger societal, and environmental factors (e.g. education, employment), as well as by demographics such as age, gender, and ethnicity [1]. In recent decades, there has been increased recognition of the importance of the socioeconomic determinants of mental health and socioeconomic inequalities in mental health [2-4]. The association between poverty and mental health appears to be universal, occurring in all societies irrespective of their level of development [5]. Nevertheless, there are differences in socioeconomic health inequalities between countries connected to their political tradition or system [6], as well as their level of economic development [7,8]. The greater the gap between the rich and the poor, the greater the differences in mental health [9].

Until 1991, Slovenia was a socialist country (part of Yugoslavia). Its leading principle was equality (equal opportunities, availability, and outcomes), so studies on socioeconomic and health inequalities would be marked by potential biases. From 1991 to 2004, the Slovenian economy had to face a complete change of markets and institutions, as well as various forms of transitions and integration connected to the European Union [10]. After its independence, Slovenia preserved elements of its past egalitarian political system to a certain extent with elements of solidarity – such as universal access to health care and to the social and educational systems. In 2009 Slovenia was confronted by the financial and economic crisis, which is still progressively weakening the material welfare of households and increasing the risk of poverty. As in other countries, the consequences can be seen in both the drop in socioeconomic position and in the increase of poverty. Nevertheless, in 2013 Slovenia had the lowest rates of social inequalities of all the EU members, when considering various welfare indicators, social exclusion, and income inequality indicators [11].

A possible indicator of more implicit differences might be the inequalities apparent in self-assessed health. In fact, we have been observing for the past 30 years that one’s educational level is an important determinant for the self-assessed health of the Slovenian adult population [12]. In Slovenia, education is also strongly connected to depression in adults of both genders [13,14], even more strongly than in the other 22 countries included in the European Social Survey [14]. Education is also the most important factor connected to gender differences in the level of depression. Its influence is substantially higher among women in Slovenia [13]. Usually the differences in mental health across the genders are not only substantiated by biological and psychological, but also by socially and culturally burdensome factors [14-16].

Although studies on mental health inequalities mainly focus on the adult population, examining this topic in the earlier stages of life is becoming increasingly common [17-22]. A review of the findings (of studies published in English or German between 1990 and 2011) showed a clear relationship between socioeconomic deprivation and mental health problems in childhood and adolescence [22]. Therefore, the correlation between socioeconomic inequalities and common mental health problems not only occurs in adulthood, but also during childhood and adolescence. An examination of various indicators of socioeconomic status (SES) revealed that a low household income and low parental education were the strongest predictors of mental health problems in children and adolescents. Some [23] also emphasize the importance of the adolescents’ personal social position.

There are several theoretical approaches to explaining the underlying association between socioeconomic position and mental health problems. The social causation hypothesis implies that the stress associated with a low social position contributes to the development of mental disorders, while the social selection hypothesis suggests that genetically predisposed individuals drift down to such a position [24]. Contemporary theories of change in youth and during the transition to adulthood are also important in understanding the socioeconomic impact on self-assessed health among adolescents. The conditions while growing up have been changing. An ever-increasing number of diverse ambivalences are also increasing the social and psychological vulnerability of youth [25,26]. According to these theories, expectations and fear of the future influence the subjective well-being more than the past or present [27].

In Slovenia, there have been some studies on health-related quality of life and health issues among adolescents and students [28-31], but we lack survey data on children and adolescent mental health inequalities. The purpose of this study is to examine mental health inequalities in Slovenian 15-year-old adolescents. This study’s specific aims are to explore the associations between self-assessed mental health and self-assessed financial position, the Family Affluence Scale, parents’ occupations, family type, the number of close friends, and the type of school. We hypothesize that girls experience poorer mental health than boys, and that adolescents with a lower socioeconomic position (SEP) have poorer mental health than those with a higher SEP.

## Methods

### Description of the study, sample, and data set

#### Study

The data was obtained from the Slovenian sample of the World Health Organization collaborative international study on Health Behavior in School-Aged Children (HBSC) conducted in March 2010. The aim of the HBSC study was to gain insight into and increase the understanding of school-aged (11, 13, and 15 years old) children’s health behavior, health outcome, and social context. A detailed description of the aims and theoretical framework of the study can be found elsewhere [32,33]. The study was approved by the Slovenian Medical Research Ethics Committee. We also obtained written, informed consent from the parents of the 15-year-olds selected in the sample.

#### Sample

HBSC is a cross-sectional study that uses the school-class as a sampling unit, randomly selected at the national level and stratified by the type of secondary school. This study focuses only on data pertaining to 15-year-olds, since this was the only age group who answered additional questions on mental health. Participation in the study was voluntary for both schools and pupils. The selected sample of the population consisted of 2,329 15-year-olds; 1,815 pupils finally participated in the study (77.93% sample realization).

#### Data set

The data was collected in schools (by education counsellors and teachers) using an anonymous international questionnaire with a total of 63 questions. The database, examined and prepared according to international methodology, was then communicated to the HBSC Data Bank (at the Norwegian Social Science Data Services [33]). Further analyses were carried out on the basis of the consolidated Slovenian database.

### Instruments

#### Mental health (MH) indicators

##### KIDSCREEN-10

The mental health of the adolescents was assessed using the KIDSCREEN-10 (Health-Related Quality of Life Questionnaire–HRQoL). KIDSCREEN instruments assess children’s and adolescents’ subjective health and well-being. The answers to the questions are classified in a 5 point scale (1–not at all or never; 5–all the time or always). A higher scale value indicates a better health-related quality of life, while a lower value indicates a poor health-related quality of life. The respondents were classified into three groups according to the results, namely those with a low, average, and high health-related quality of life.

##### Feelings of depression

In order to assess the occurrence of feelings of depression, we asked the following question: *Has there been a period in the past year when you felt so sad almost every day for a period of two or more weeks in a row that it made you stop doing things you normally do*? The answer was either positive or negative. Using this answer, we cannot talk about the presence of depressive disorders, but only about an increased risk of it. In our paper, we use the term “feelings of depression” with the described meaning.

##### Life satisfaction

We employed a Cantrill ladder, where adolescents were asked to indicate the step of the ladder where they would place their lives at present. The top of the ladder is 10 and indicates *“the best possible life for you”* while the bottom of the ladder is 0 and indicates *“the worst possible life for you.”* We then divided adolescents into two groups; those who were satisfied with their lives (indicated by a score of 6 or more on the scale), and those who were dissatisfied with their lives (less than 6).

##### The Strengths and Difficulties Questionnaire (SDQ)

The Strengths and Difficulties Questionnaire (SDQ) is a brief behavioral screening questionnaire about 3-16 year olds. It exists in several versions and we used 20 questions referring to four aspects (subscales) of mental health: emotional and behavioral problems, hyperactivity, and difficulties in interaction with peers. Based on the answers, each question is graded on a scale (0–not true; 1–somewhat true; 2–completely true). The overall result is the sum of the results of the individual subscales. The higher the score, the greater the probability of mental health difficulties. We divided the participants into three groups according to the number of points achieved; a normal result–a low probability of difficulties; cut-off result–the probable presence of clinically significant difficulties; and increased result–an increased probability of the presence of clinically significant difficulties.

The validity of our mental health measures and the cut point criteria have been examined in several previous studies [33-35].

#### Socioeconomic position (SEP) indicators

In our paper, we include gender and several variables that measure adolescents’ socioeconomic position: self-assessed financial position, the FAS (family affluence scale), parents’ occupation, family type, the number of close friends, and the school type. These variables have been roughly divided into two categories: the socioeconomic status of the adolescent’s family, and the adolescent’s personal social position [23].

#### The socioeconomic status of the adolescent’s family

##### Self-assessed financial position

Perceived material welfare is a single item question where adolescents answer how well their family is doing financially (in their opinion) on a scale from 1 to 5 (1–very well; 5–not well at all). For the purpose of the analysis, we combined the answers into the following three categories: above average (very well and rather well), average, and below average (not too well and not well at all) material welfare in the adolescent’s family.

##### Family Affluence Scale (FAS)

The Family Affluence Scale (FAS) measures family wealth. The included items refer to car ownership, bedroom occupancy, holidays, and home computers, and measure the material conditions of the households. A composite score is calculated for each young person, which is then split according to international criteria [36,37] into those with a low, medium, and high FAS. Given that, we established that the prescribed classification is not consistent with socioeconomic stratification in Slovenia, because more than half of the children were classified in the high socioeconomic class [38]. Given the fact that some authors decided on a different classification when the classification was not consistent with the country’s situation [39], we have used the following classification that reflects the Slovenian socioeconomic stratification in a more accurate way:

• FAS 1 (total of 0 to 4) indicates a low socioeconomic status,

• FAS 2 (total of 5 and 6) indicates a middle socioeconomic status,

• FAS 3 (total of 7) indicates a high socioeconomic status.

##### Occupational status

We formed variables from the parent’s occupational status. The answers were categorized into three groups: both parents unemployed, one parent unemployed, and both parents employed.

##### Family type or family structure

Family structure: the items are focused on a set of questions about household composition. The answers have been categorized into three groups: living with both parents, a single-parent family, and a reconstituted family.

##### The adolescent’s personal social position (PSP)

Personal social position was measured using the number of really close friends (answers were categorized into three possible answers: no friends, 1-2 friends, and 3 or more friends) and by school type. There are three types of secondary school in Slovenia: industrial and crafts schools (3 years or less, where students obtain a profession when they finish the studies), technical and related schools (4 years, students receive a vocational degree) and the most general schools known as ‘gymnasiums’ –the European equivalent to ‘high schools’ –(4 years, students don’t receive a vocational degree yet, but can proceed directly to college/university studies). Secondary education is provided equally for everyone upon the completion of nine years of primary education, based on individual achievement in primary education and the highest level of achievement is required to attend gymnasiums.

### Statistical method and analyses

In order to calculate the association between variables, we used the Chi-square test (*X*^2^) and calculated the unadjusted odds ratios that enable us to infer from the sample to the population. Later, we applied multiple logistic regression and multivariate analysis of variance (MANOVA) for detecting the differences in the mental well-being of adolescents according to their SEP. Two logistic regressions were used for assessing the relationship between two binary dependent variables (feelings of depression and low life satisfaction) and the SEP indicators. For the other two numerical dependent variables, KIDSCREEN-10 and SDQ, a MANOVA analysis was applied. The assumptions for the MANOVA analysis were checked in advance (i.e., a test of multivariate normality, the homogeneity of variances). As the original variables for life satisfaction, KIDSCREEN-10 and SDQ were not measured on the same scale and were not normally distributed, a Box-Cox transformation, followed by a Z-score calculation, were used in order to make the data as suitable as possible for the analysis. The last two variables (KIDSCREEN-10 and SDQ), which passed all of the assumptions, were actually used in the MANOVA model. The level of significance was set at *α* = 0.05. All analyses were conducted in Stata 11.2 or IBM SPSS 19.

## Results and discussion

### Results

#### Sample description, including socioeconomic position

Table 1 shows the distribution of dependent and independent indicators in the Slovenian sample.

**Table 1:** Sample description

**Variable**		**n (%)**
**Gender**	Boy	914 (50.4)
	Girl	901 (49.6)
**KIDSCREEN-10**	Low	733 (41.0)
	Average	722 (40.4)
	High	332 (18.6)
**Feelings of depression**	Yes	623 (35.1)
	No	1151 (64.9)
**Life satisfaction (LF)**	High LF	1153 (84.9)
	Low LF	268 (15.1)
**SDQ**	Normal	1422 (80.1)
	Borderline	209 (11.8)
	Increased	143 (8.1)
**Perceived material welfare**	Above average (fine, very good)	1064 (59.1)
	Average	572 (31.8)
	Below average or bad	163 (9.1)
**FAS**	High	535 (29.9)
	Middle	909 (50.8)
	Low	344 (19.3)
**Parent’s employment**	Both employed	1328 (74.0)
	One unemployed	410 (22.8)
	Both unemployed	58 (3.2)
**Family type**	Classic	1439 (80.4)
	Single-parent	238 (13.3)
	Reconstituted	113 (6.3)
**Number of friends (both sexes)**	3+ friends	1574 (87.4)
	1-2 friends	210 (11.7)
	0 friends	16 (0.9)
**School type**	Gymnasiums (general)	772 (43.4)
	Technical & related	641 (36.1)
	Industrial & craft	364 (20.5)

Table 2 provides the distribution and association between gender and socioeconomic position. More than half of the adolescents perceived their family’s material welfare as very good or fine. Girls perceived their family’s material welfare worse than boys. According to FAS, most of the participants (50.8%) were classified in the middle FAS. There were no significant differences between genders. The majority of adolescents’ parents are employed and only 3.2% of the adolescents’ families have both parents unemployed. There were no significant differences between genders. The majority (80.4%) of adolescents lived in a classic family with both parents; there are significant differences between genders though. Most adolescents had three or more friends; less than 1% had no friends. There were no significant differences between genders. Most of the adolescents attended gymnasiums, followed by technical and related schools, while the fewest attended industrial and crafts schools (Table 2).

**Table 2:** Socioeconomic position according to gender

**Variable**		**Girls n (%)**	**Boys n (%)**	**χ**^**2**^**(p value)**
**Perceived material welfare**	Above average (fine, very good)	501 (55.9)	563 (62.4)	8.62 *(0.013)*
	Average	302 (33.7)	270 (29.9)	
	Below average or bad	93 (10.4)	70 (7.7)	
**FAS**	High	255 (28.5)	280 (31.3)	5.61 (0.060)
	Middle	447 (50.1)	462 (51.6)	
	Low	191 (21.4)	153 (17.1)	
**Parent’s employment**	Both employed	658 (73.3)	670 (74.6)	0.54 (0.763)
	One unemployed	209 (23.3)	201 (22.4)	
	Both unemployed	31 (3.4)	27 (3.0)	
**Family type**	Classic	733 (82.3)	706 (78.5)	7.41 *(0.025)*
	Single-parent	99 (11.1)	139 (15.5)	
	Reconstituted	59 (6.6)	54 (6.0)	
**Number of friends (both sexes)**	3+ friends	770 (86.0)	804 (88.8)	3.42 (0.181)
	1-2 friends	117 (13.1)	93 (10.3)	
	0 friends	8 (0.9)	8 (0.9)	
**School type**	Gymnasiums (general)	467 (52.5)	305 (34.4)	128.8 *(p < 0.001)*
	Technical & related	336 (37.8)	305 (34.4)	
	Industrial & craft	86 (9.7)	278 (31.3)	

#### Mental health according to gender

The great majority of 15-year-old Slovenian boys had a high quality of life, they did not have any feelings of depression, had high life satisfaction and a normal score on the SDQ scale. On the contrary, girls had a statistically significant lower quality of life, lower life satisfaction, and a higher score on the SDQ scale. They also had more feelings of depression than boys (Table 3).

**Table 3:** Mental health indicators according to gender

**Variable**	**Category**	**Girls**	**Boys**	**χ**^**2**^**or t, (p value)**	**Odds Ratio (95% CI)**
		**n (%)**	**n (%)**		
**KIDSCREEN-10**	Low	421 (47.4)	312 (34.7)	χ^2^ = 44.7 *(p < 0.001)*	1.70 (1.40–2.06)
	Average	349 (39.3)	373 (41.5)		
	High	118 (13.3)	214 (23.8)		1.00
	Total	xˉ=36.23	xˉ=38.33	t = 6.76 *(p < 0.001)*	-
		SD = 5.58	SD = 6.62		
**Feelings of depression**	Yes	407 (46.5)	216 (24.1)	χ^2^ = 97.7 *(p < 0.001)*	2.74 (2.23–3.37)
	No	469 (53.5)	682 (75.9)		1.00
**Life satisfaction (LF)**	High LF	726 (81.9)	787 (87.9)	χ^2^ = 12.5 *(p < 0.001)*	1.00
	Low LF	160 (18.1)	108 (12.1)		1.61 (1.22–2.11)
	Total	xˉ=7.21	xˉ=7.58	t = 4.26 *(p < 0.001)*	-
		SD = 1.89	SD = 1.70		
**SDQ**	Normal	688 (77.5)	734 (82.8)	χ^2^ = 8.2 *(p = 0.017)*	1.00
	Borderline	117 (13.2)	92 (10.4)		1.40 (1.10–1.80)
	Increased	83 (9.3)	60 (6.8)		
	Total	xˉ=11.66	xˉ=10.41	t = 5.02 *(p < 0.001)*	-
		SD = 5.22)	SD = 5.27)		

Table 4 presents the results of two logistic regression models. The first model for lower life satisfaction was valid and correctly classified in 85.3% of the cases. Three independent variables made a statistically significant contribution to the model: gender; perceived material (average and bad vs. very good) welfare and school type. All other SEP variables (family type, parents’ occupation, number of friends, FAS) did not make a statistically considerable contribution to the model. The higher odds for low life satisfaction were assessed in girls (1.5-times higher compared to boys), 15-year-olds who perceived their families’ material welfare as worse (4-times higher compared to those who perceived it better), and those attending industrial and crafts schools (2-times higher than those attending gymnasiums).

**Table 4:** Results of a logistic regression of predictors of life dissatisfaction and feelings of depression (n = 1644)

**Variable**	**Comparison**	MODEL 1: Low life satisfaction*		MODEL 2: Feelings of depression: yes**	
		**Odds ratio (95% CI)**	**P > |z|**	**Odds ratio (95% CI)**	**P > |z|**
**Gender**	Girl vs. boy	1.55 (1.12–2.15)	*0.008*	2.57 (2.04–3.24)	*< 0.001*
**Family type**	Single-parent vs. classic	1.46 (0.97–2.21)	0.072	1.02 (0.73–1.42)	0.896
	Reconstituted vs. classic	1.02 (0.56–1.86)	0.943	1.25 (0.82–1.92)	0.301
**Perceived material welfare**	Fine vs. very good	1.31 (0.79–2.17)	0.290	1.10 (0.80–1.51)	0.569
	Average vs. very good	1.78 (1.07–2.97)	*0.027*	1.07 (0.76–1.50)	0.708
	Bad/worse vs. very good	4.12 (2.28–7.47)	*<0.001*	1.30 (0.81–2.07)	0.275
**Number of friends (both sexes)**	None vs. 3+	1.66 (0.39–7.16)	0.496	1.94 (0.59–6.44)	0.277
	1-2 vs. 3+	1.14 (0.73–1.79)	0.556	0.88 (0.63–1.24)	0.475
**School type**	Technical vs. gymnasiums	1.260 (0.89–1.79)	0.190	1.22 (0.96–1.56)	0.106
	Industrials vs. gymnasiums	2.26 (1.47–3.49)	*<0.001*	1.14 (0.81–1.59)	0.456
**Parents’ occupation**	Only one employed vs. both employed	1.01 (0.71–1.46)	0.937	0.90 (0.68–1.18)	0.440
	Both unemployed vs. both employed	0.52 (0.21–1.32)	0.170	1.39 (0.75–2.59)	0.299
**FAS**	Low vs. high	1.39 (0.88–2.20)	0.160	1.15 (0.82–1.61)	0.427
	Middle vs. high	1.10 (0.75–1.62)	0.614	1.09 (0.85–1.41)	0.496

*n = 1644, p = 0.222, R^2^ = 0.126, AUC = 0.75.

**n = 1644, p = 0.227, R^2^ = 0.086, AUC = 0.69.

The second model for feelings of depression was also valid and correctly classified in 69.2% of cases. Only gender made a statistically significant contribution to the model, while SEP variables did not. In comparison to boys, girls have 2.5-times greater odds of suffering from feelings of depression (p = <0.001).

The results of the MANOVA analysis (Table 5) showed gender, perceived material welfare, number of friends, and school type as statistically significant. The explained percentage of variance is small (KIDSCREEN = 8.04%, p < 0.001; SDQ = 6.01%, p < 0.001), yet statistically significant.

**Table 5:** Results of the MANOVA for KIDSCREEN-10 and SDQ

**Variable**	**Comparison**	**KIDSCREEN-10**		**SDQ**		**Df**	**F**	**Wilks’ λ (p)**
		**Coef.**	**P > |t|**	**Coef.**	**P > |t|**			
**Gender**	Girl vs. boy	-0.28	*0.001*	-0.27	*0.001*	1	7.33	0.976 *(<0.001)*
**Family type**	Single-parent vs. classic	-0.09	0.205	-0.13	0.079	2	1.46	0.997 (0.211)
	Reconstituted vs. classic	-0.16	0.095	-0.14	0.167			
**Perceived material welfare**	Fine vs. very good	-0.14	*0.036*	-0.15	*0.030*	3	7.77	0.973 *(<0.001)*
	Average vs. very good	-0.34	*<0.001*	-0.28	*<0.001*			
	Bad/worse vs. very good	-0.49	*<0.001*	-0.54	*<0.001*			
**Number of friends (both sexes)**	None vs. 3+	-0.59	*0.021*	-0.59	*0.023*	2	6.23	0.985 *(<0.001)*
	1-2 vs. 3+	-0.31	*<0.001*	-0.22	*0.003*			
**School type**	Technical vs. gymnasiums	0.12	*0.018*	-0.09	0.096	2	11.30	0.974 *(<0.001)*
	Industrials vs. gymnasiums	0.18	*0.006*	-0.25	*<0.001*			
**Parent’s employment**	Only one employed vs. both employed	-0.01	0.803	0.07	0.273	2	0.71	0.998 (0.585)
	Both unemployed vs. both employed	0.09	0.516	0.19	0.889			
**FAS**	Low vs. high	-0.08	0.309	-0.08	0.296	2	0.47	0.999 (0.760)
	Middle vs. high	0.00	0.949	0.00	0.998			

A lower KIDSCREEN-10 and higher SDQ score are significantly more probable for female adolescents, for adolescents who have assessed their financial position as less than very good (the probabilities increase as the assessment decreases), and for adolescents with no or 1-2 really close friends. Adolescents who attended an industrial and craft or technical and related school program tend to have a higher probability for higher KIDSCREEN-10 scores than their peers attending a gymnasium. On the other hand, regarding SDQ scores, no significant differences have been found for technical and related school programs in comparison to gymnasiums; however, there is a significant difference between industrial and crafts and gymnasiums; adolescents from industrial and craft school program have increased probabilities of higher SDQ scores.

## Discussion

Findings from this study imply that gender, perceived material welfare, number of friends, and school type somewhat determine certain mental health indicators. Girls achieved a lower score for the mental health indicators than boys. Adolescents with a lower socioeconomic position have poorer mental health than those with a higher SEP, but not all socioeconomic variables are associated with all mental health indicators. However, the results were mostly in line with our hypothesis.

Gender is an important predictor of adolescents’ mental health. Among those Slovenian 15-year-old adolescents who reported problems with mental health, there was a significantly larger number of girls. The finding is consistent with similar studies that reported poorer mental health among girls [39-43]. The differences in mental health across genders are not only substantiated by individual factors such as biological (neurohormonal or genetic) and psychological (reaction to the stressors), but also by socially and culturally burdensome factors such as socioeconomic position, education, ethnicity [15,16], gender culture, gender order, and gender regime [44]. The gender culture in this postmodern society often exposes women to discriminatory factors (such as lack of educational, work, and career opportunities, and an underestimation of women’s roles and activities), and to new demands which are related to bodily, dietary, and health regimes [13]. In Slovenia, not only women, but also young adolescent girls are submitted to some discriminatory factors and demands. Girls in Slovenia are more successful in the educational process and attain higher educational levels than boys, however they receive lower wages than men and few take on important positions [45]. Moreover, according to Šribar [46], girls in Slovenia develop low self-esteem more frequently than boys.

A study about adults on gender differences in depressive symptoms in Slovenia showed that education was the most important differing factor. With increasing education the rate of depression in Slovenia decreases more rapidly in women than in men [13]. In another study among the student population in Slovenia, there were no statistically significant differences between men and women in the prevalence of depression [30].

The examination of various indicators of socioeconomic position revealed that perceived material welfare (an indicator of the socioeconomic position of the adolescent’s family) and the number of friends and school type (indicators of the adolescent’s personal social position) were (beside gender) the strongest predictors of mental health problems among Slovenian 15-year-old adolescents.

Self-assessed financial position has been identified as an important factor for mental health status by several studies [22,34,42,43,47,48]. Likewise, our results show that perceiving the familial financial position as poor decreases adolescent's life satisfaction and increases the risk of mental health problems. Adolescents from socioeconomically disadvantaged families are probably deprived in many ways in comparison to peers from socioeconomically advantaged families (holiday destinations, possession of high-tech devices, clothing, etc.), which could influence their perceived position among peers. On the other hand, in contrast to some other studies’ findings [49,50], FAS and parents’ occupation have not been found to be an important factor.

Moreover, having no, or only 1-2, really close friends were found to be risk factors for mental health problems and lower life quality. These results were consistent with some other studies [34,39,51] that suggested a lack of social support as a possible contributor to health inequalities. Also, Goodman et al. [52] found that adolescents’ subjective social status is an important predictor of their health. Peer relationships are very important in this developmental stage; quality and closeness in friendship are also connected to the experience of self and identity [53].

The study found that the school type played an important role in relation to adolescents’ mental health. Adolescents attending industrial or crafts and technical or related school programs reported higher KIDSCREEN-10 levels (or higher quality of life) and worse mental health assessments (higher SDQ scores and lower life satisfaction) than gymnasiums. Possible explanations still need to be explored. Magklara et al. [18] report that low academic achievement is linked to poor psychological health. It could be that the school achievement is more important for gymnasium school pupils than it is for those in industrial or crafts and technical or related schools, therefore contributing to lower KIDSCREEN-10 scores. Some authors also report that a poor school environment increases the odds of lower self-rated health, multiple health complaints, and lower life satisfaction [54]. Thus, better KIDSCREEN-10 (higher) and SDQ scores (lower) might have an underlying dimension connected to the school environment. Further analyses are needed to examine the differences in school environment according to school type.

Several theoretical approaches were established to explain the underlying association between the socioeconomic position and mental health problems. The social causation hypothesis posits that mental health problems result from socioeconomic deprivation. Individuals from socioeconomically disadvantaged families are more likely to develop mental health problems than those from socioeconomically advantaged ones [24]. The social selection hypothesis assumes that people with mental health problems drift downwards in socioeconomic position because of their mental health problems and their inability to fulfill expected role obligations [24]. Low childhood socioeconomic position was found to be associated with disadvantages in health and economic position in adulthood. In our study, we found support for the social causation hypothesis by demonstrating that socioeconomic position contributes to differences in the levels of mental health problems.

In addition, differences between adults from high and low classes in Slovenia have been increasing [38], and we can expect to see a greater impact of social inequalities on adolescents’ mental health status in the future. In 2010 and 2011, the at-risk-of-poverty rate for children exceeded the general risk of poverty for the whole population for the first time [11]. In 2011, the at-risk-of-poverty rate in Slovenia was 12.7%, 1.4 percentage points higher than in the previous year [55]. Without a fairly effective system of social transfer, the poverty rate would be much higher. There is a danger that the universal systems of public health, education, and social assistance systems might be reformed (Slovenia adopted many anti-crisis measures) in a way that increases social inequalities by substantially transferring the care for health, education, childcare, and social security from the state to individuals [28]. The World Health Organization argues that in times of economic crisis, countries tend to reduce investments in health and determinants of health, which may lead to a long-term deterioration of the population’s physical and mental health. It is thus important, when preparing political measures, to evaluate and reasonably consider their possible effects on health.

Contemporary theories of change in youth and the transition to adulthood are also important for understanding the socioeconomic impact on self-assessed mental health among adolescents. The conditions for growing up have been changing and the individualization of life courses is increasing the social and psychological vulnerability of youth [25,26]. Slovenian youths are confronted with a competitive educational system, a demanding and restrictive labor market, and a prolonged period of dependence on their parents. The transition from youth to adulthood is no longer as predictable as it was, but riskier and more uncertain. All of this could contribute to their mental health.

### Study limitations

The study includes only 15-year-olds who are enrolled in school and does not include dropouts, who might be among the most socioeconomically underprivileged. Moreover, feelings of depression were measured with only one question. In addition, mental health and socioeconomic position were based solely on adolescents’ self-reporting. Our study doesn’t include other important SEP variables for adolescents and their parents. According to other studies [22], parents’ education, household income, and parents’ mental health status (a history of mental health problems) would be of special interest.

## Conclusions

Personal social position and the perceived material welfare of the family are associated with mental health problems in Slovenian 15-year-olds. According to the results of this study, a lower socioeconomic position, few friends or none at all, and a less demanding type of school were all found to be a risk factor for mental health problems. These results are particularly relevant for understanding mental health inequalities in Slovenia and further discussions and policy actions. These findings emphasize the need for intervention to reduce poverty and social isolation in children and adolescents. Special focus should be given to the least advanced secondary schools (industrial and craft).

## Competing interests

The authors declare no competing interests in relation to this paper.

## Authors’ contributions

HJK was responsible for the conception and design of the study and data collection, contributed to the statistical analyses, and had the main responsibility for writing the manuscript. JŽ contributed to the statistical analysis and participated in writing the manuscript and interpreting the results. AK performed the statistical analysis and helped in writing the manuscript and interpreting the results. JZ contributed to the statistical analysis and made critical comments that helped in the interpretation of the results and the final writing of the paper. TA made critical comments on the paper and helped in the interpretation of the results. All the authors have read and approved the final manuscript.

## References

[B1] Goldie I Public mental health today: a handbook 2010 Brighton: Pavilion Publishing Ltd

[B2] Marmot M Bell R Fair society, healthy lives Public Health 2012 126 Suppl 3 S4 S10 22784581 10.1016/j.puhe.2012.05.014

[B3] Fisher M Baum F The social determinants of mental health: implications for research and health promotion Aust N Z J Psychiatry 2010 44 1057 1063 10.3109/00048674.2010.509311 20973623

[B4] Fryers T Melzer D Jenkins R Social inequalities and the common mental disorders: a systematic review of the evidence Soc Psychiatry Psychiatr Epidemiol 2003 38 229 237 10.1007/s00127-003-0627-2 12719837

[B5] WHO Promoting Mental Health: Concepts, Emerging Evidence, Practice: Summary Report/A Report from the World Health Organization, Department of Mental Health and Substance Abuse in collaboration with the Victorian Health Promotion Foundation (VicHealth) and the University of Melbourne 2004 Geneva: World Health Organization

[B6] Vicente N Leiyu S The political context of social inequalities and health Soc Sci Med 2001 52 481 491 10.1016/S0277-9536(00)00197-0 11330781

[B7] Wroblewska W Womens health status in Poland in the transiton to a market economy Soc Sci Med 2002 54 707 726 10.1016/S0277-9536(01)00104-6 11999488

[B8] Demirchyan A Petrosyan V Thompson ME Gender differences in predictors of self-rated health in Armenia: a population-based study of an economy in transition Int J Equity Health 2012 11 67 23151068 10.1186/1475-9276-11-67 PMC3544611

[B9] Picket K Oliver J Wilkinson R Income inequality and the prevalence of mental illness: a preliminary international analysis J Epidemiol Community Health 2006 60 646 647 10.1136/jech.2006.046631 16790839 PMC2652881

[B10] Črnak Meglič A Children and youth in the transitional society: analysis of the situation in Slovenia 2006 Maribor: Aristej

[B11] Urad RS za makroekonomske analize in razvoj Development report 2013 http://www.umar.gov.si/fileadmin/user_upload/publikacije/dr/13/A_por13_s.pdf

[B12] Malnar B Kurdija S Trends in subjective health assessment between 1981 and 2011 as an indicator of persistent social inequalities Zdrav Var 2012 51 11 20

[B13] Kamin T Berzelak N Ule M The influence of education on differences in depressive symptoms between men and women in Slovenia Zdrav Var 2011 51 33 42

[B14] Von dem Knesebeck O Pattyn E Bracke P Education and depressive symptoms in 22 European countries Int J Public Health 2010 56 107 110 20963468 10.1007/s00038-010-0202-z

[B15] Lubotsky LB, Becker MA A public health perspective of womens’ mental health 2010 New York, London: Springer

[B16] Annandale E Hunt K Gender inequalities in health 2000 Philadelphia: Open University Press

[B17] Davis E Sawyer MG Lo SK Priest N Wake M Socioeconomic risk factors for mental health problems in 4-5-Year-Old Children; Australian population study Acad Pediatr 2010 10 41 47 10.1016/j.acap.2009.08.007 20129480

[B18] Magklara K Skapinakis P Niakas D Bellos S Zissi A Stylianidis S Mavreas V Socioeconomic inequalities in general and psychological health among adolescents: a cross-sectional study in senior high schools in Greece Int J Equity Health 2010 9 3 20181002 10.1186/1475-9276-9-3 PMC2837664

[B19] Amone-P’Olak K Burger H Ormel J Huisman M Oldehinkel AJ Verhulst FC Socioeconomic position and mental health problems in pre- and early-adolescents: the trails study Soc Psychiatry Psychiatr Epidemiol 2009 44 231 238 10.1007/s00127-008-0424-z 18714424

[B20] Dashiff C DiMicco W Myers B Sheppard K Poverty and adolescent mental health J Child Adolesc Psychiatr Nurs 2009 22 23 32 10.1111/j.1744-6171.2008.00166.x 19200289

[B21] McLaughlin KA Breslau J Green JG Lakoma MD Sampson NA Zaslavsky AM Childhood socio-economic status and the onset, persistence, and severity of DSM-IV mental disorders in a US national sample Soc Sci Med 2011 73 1088 1096 10.1016/j.socscimed.2011.06.011 21820781 PMC3191493

[B22] Reiss F Socioeconomic inequalities and mental health problems in children and adolescents: a systematic review Soc Sci Med 2013 90 24 31 23746605 10.1016/j.socscimed.2013.04.026

[B23] Koivusilta LK Rimpelä AH Kautiainen SM Health inequality in adolescence: does stratification occur by familial social background, family affluence or personal social position? BMC Public Health 2006 6 110 10.1186/1471-2458-6-110 16643660 PMC1479325

[B24] Murali V Oyebode F Poverty, social inequality and mental health Adv Psychiatr Treat 2004 10 216 10.1192/apt.10.3.216

[B25] Ule M Za vedno mladi? Socialna psihologija odraščanja 2008 Ljubljana: Fakulteta za družbene vede

[B26] Ule M Rener T Mencin-Čeplak M Tivadar B Socialna ranljivost mladih 2000 Maribor: Aristej

[B27] Kurdija S Ule M Živoder A A generation presents itself: “teenagers” satisfaction and processing of the social reality in Slovenia Teorija Praksa 2011 48 1486 1504

[B28] Ule M Tivadar B Kurdija S Rajšp S Socialnoekonomski položaj študentov v Sloveniji 2008 Ljubljana: Univerza v Ljubljani, Fakulteta za družbene vede, Center za socialno psihologijo

[B29] Ule M Kuhar M Kakovost življenja otrok in mladostnikov v Sloveniji: Študija osipništva 2003 Ljubljana: FDV

[B30] Klemenc-Ketiš Z Hladnik Ž Rotar-Pavlič D Post M Kersnik J Self-reported chronic conditions in student populations in Slovenia Zdrav Vestn 2010 79 31 41

[B31] Lavrič M Mladina 2010: Družbeni profil mladih v Sloveniji 2011 Maribor: Aristej

[B32] Currie C, Nic Gabhainn S, Godeau E, Roberts C, Smith R, Currie D, Picket W, Richter M, Morgan A, Barnekow V Inequalities in young people’s health: HBSC international report from the 2005/06 survey 2008 Copenhagen, Denmark: WHO, Regional Office for Europe (health policy for Children and Adolescents, No. 5)

[B33] Currie C, Zanotti C, Morgan A, Currie D, Looze M de, Roberts C, Samdal O, Smith ORF, Barnekow V Social determinants of health and well-being among young people. Health Behaviour in School- aged children (HBSC) study; International report from the 2009/2010 survey 2012 Copenhagen: WHO, Regional Office for Europe (health policy for Children and Adolescents, No. 6)

[B34] Ravens-Sieberer U Erhart M Gosch A Wille N The European KIDSCREEN Group Mental health of children and adolescents in 12 European Countries – results from the European KIDSCREEN study Clin Psychol Psychother 2008 15 154 163 10.1002/cpp.574 19115436

[B35] Goodman R Meltzer H Bailey V The strengths and difficulties questionnaire: a pilot study on the validity of the self-report version Eur Child Adolesc Psychiatry 1998 7 125 130 10.1007/s007870050057 9826298

[B36] Boyce W Torsheim T Currie C Zambon A The family affluence scale as a measure of national wealth: validation of an adolescent self-report measure Soc Indic Res 2006 78 473 487 10.1007/s11205-005-1607-6

[B37] Currie C Molcho M Boyce W Holstein B Torsheim T Richter M Researching health inequalities in adolescents: the development of the Health Behaviour in School-Aged Children (HBSC) family affluence scale Soc Sci Med 2008 66 1429 1436 10.1016/j.socscimed.2007.11.024 18179852

[B38] Artnik B Drev A Drglin Z Fajdiga VT Gabrijelčič MB Gregorič M Hočevar T Jeriček HK Koprivnikar H Kovše K Mihevc BP Pucelj V Rok MS Roškar S Scagnetti N Zupančič T Neenakosti v zdravju in z zdravjem povezanih vedenjih slovenskih mladostnikov (Inequalities in health and health behavior among Slovenian adolescents) 2008 Ljubljana: Inštitut za varovanje zdravja Republike Slovenije

[B39] Holstein BE Currie C Boyce W Damsgaard MT Gobina I Kökönyei G Hetland J de Looze M Richter M Due P HBSC Social Inequalities Focus Group Socio-economic inequality in multiple health complaints among adolescents: international comparative study in 37 countries Int J Public Health 2009 45 260 270 19639254 10.1007/s00038-009-5418-4

[B40] Chhabra GS Sodhi MK Factors contributing to psycho-social Ill-health in male adolescents Online J Health Allied Scs 2011 10 2

[B41] Bisegger C Cloetta B Von Rueden U Abel T Ravens-Sieberer U European Kidscreen Group Health-related quality of life: gender differences in childhood and adolescence Soz Preventivmed 2005 50 281 291 10.1007/s00038-005-4094-2 16300172

[B42] Levin KA Dallago L Currie C The association between adolescent life satisfaction, family structure, family affluence and gender differences in parent–child communication Soc Indic Res 2012 106 287 305 10.1007/s11205-011-9804-y

[B43] Piko FB Hamvai C Parent, school and peer-related correlates of adolescents’ life satisfaction Child Youth Serv Rev 2010 32 1479 1482 10.1016/j.childyouth.2010.07.007

[B44] Pfau-Effinger B Gender cultures and the gender arrangement: a theoretical framework for cross-national gender research Innovation 1998 11 147 162

[B45] Dan žensk 2012, SURS http://www.stat.si/novica_prikazi.aspx?id=4548

[B46] Šribar R Ule M Status and social opportunities of the girl child: expert study 2008 Ljubljana: University of Ljubljana, Faculty of Social Sciences

[B47] Hölling H Kurth BM Rothenbergerer A Becker A Schlack R Assessing psychopathological problems of children and adolescents from 3 to 17 years in a nationwide representative sample: results of the German health interview and examination survey for children and adolescents (KIGGS) Eur Child Adolesc Psychiatry 2008 17 34 41 10.1007/s00787-008-1004-1 19132302

[B48] Proctor CL Linley PA Maltby J Youth life satisfaction: a review of the literature J Happiness Stud 2009 10 583 630 10.1007/s10902-008-9110-9

[B49] Starfield B Riley AW Witt WP Robertson J Social class gradients in health during adolescence J Epidemiol Community Health 2002 56 354 361 10.1136/jech.56.5.354 11964432 PMC1732142

[B50] Von Rueden U Gosch A Rajmil L Bisegger C Ravens-Sieberer U The European KIDSCREEN group Socioeconomic determinants of health related quality of life in childhood and adolescence: results from a European study J Epidemiol Community Health 2006 60 130 135 10.1136/jech.2005.039792 16415261 PMC2566139

[B51] De Matos M Gaspar M Simones C Batista-Fouget J Cottraux J Personal and social factors associated with the perception of health and the perception of happiness in a nonclinical adolescent population Encéphale 2010 36 39 45 10.1016/j.encep.2008.08.002 20159195

[B52] Goodman E Adler NE Kawachi I Frazier AL Huang B Colditz GA Adolescents’ perceptions of social status: development and evaluation of a new indicator Pediatrics 2001 108 E31 10.1542/peds.108.2.e31 11483841

[B53] Hall-Lande JA Eisenberg ME Christenson SL Neumark-Sztainer D Social isolation, psychological health, and protective factors in adolescence Adolescence 2007 42 265 286 17849936

[B54] Simetin IP Kuzman M Franelic IP Pristas I Benjak T Dezeljin JD Inequalities in Croatian pupils’ unhealthy behaviors and health outcomes: role of school, peers and family affluence Eur J Public Health 2011 21 122 128 10.1093/eurpub/ckq002 20159771

[B55] Intihar S Income and poverty indicators, Slovenia – provisional data 2011 http://www.stat.si/eng/novica_prikazi.aspx?id=4177

